# Proteomic profiling of high purity extracellular vesicle subtypes from megakaryoblastic leukemia cells provides insights into myeloproliferative neoplasm biology

**DOI:** 10.20517/evcna.2026.38

**Published:** 2026-09-02

**Authors:** Xiaogang Zhang, Sarita Sehra, Cynthia Timmers, Tony Chadderton, Matthew Stubbs, Xiaowei Xu

**Affiliations:** ^1^Department of Pathology and Laboratory Medicine, University of Pennsylvania, Philadelphia, PA 19104, USA.; ^2^Incyte Research Institute, Wilmington, DE 19803, USA.

**Keywords:** MEG-01, extracellular vesicles, subtypes, proteomics, myeloproliferative neoplasm

## Abstract

**Aim:** Extracellular vesicles (EVs) are emerging as important mediators in myeloproliferative neoplasms (MPNs), but the characteristics of distinct EV subtypes remain unclear. This study aimed to characterize the molecular heterogeneity of MEG-01-derived large EVs (lEVs) and small EVs (sEVs).

**Methods:** lEVs and sEVs were isolated from MEG-01 cell culture medium by differential centrifugation followed by density gradient ultracentrifugation. EVs were characterized by transmission electron microscopy, nanoparticle tracking analysis, immunoblotting, and on-bead flow cytometry. Quantitative proteomic analysis was performed to compare the protein cargo of lEVs and sEVs, followed by Gene Ontology (GO) and Kyoto Encyclopedia of Genes and Genomes (KEGG) pathway enrichment analyses.

**Results:** Density gradient purification yielded high purity of lEV and sEV with distinct size, morphologies, and protein compositions. On-bead flow cytometry enabled efficient profiling of EV surface markers. Proteomic analysis revealed a shared core proteome between lEVs and sEVs, together with subtype specific protein signatures. Enrichment analyses indicated distinct molecular characteristics of the two EV populations, with sEVs showing enrichment of proteins associated with RNA-related processes and vesicle-mediated transport, whereas lEVs were enriched in proteins related to cytoskeletal organization and metabolism.

**Conclusion:** MEG-01 derived lEVs and sEVs comprise molecularly distinct subpopulations with shared and subtype specific protein cargo. The workflow established in this study provides a robust platform for high purity EV isolation and characterization, and offers insights into MEG-01 EV heterogeneity relevant to megakaryocyte biology and MPN research.

## INTRODUCTION

Myeloproliferative neoplasms (MPNs) are a group of clonal hematopoietic stem cell linked disorders characterized by excessive proliferation of one or more myeloid cell lineages. These disorders, including polycythemia vera (PV), essential thrombocythemia (ET), and primary myelofibrosis (PMF), are primarily driven by mutations in key signaling molecules such as Janus kinase 2 (JAK2), calreticulin (CALR), and myeloproliferative leukemia protein (MPL)^[1]^. Clinically, MPNs manifest with abnormal blood counts, bone marrow fibrosis, and increased risk of thrombosis and leukemic transformation^[2]^. Despite progress in elucidating genetic drivers, the complex cellular interactions and microenvironmental factors sustaining malignant hematopoiesis and disease progression remain elusive.

Extracellular vesicles (EVs) are membrane-bound nanoparticles secreted by virtually all cell types. EVs are categorized into three distinct subtypes based on their biogenesis, including exosomes, microvesicles, and apoptotic bodies^[3]^. EVs mediate intercellular communication by transferring bioactive cargos, including proteins, lipids, and nucleic acids, to recipient cells, thereby modulating their functions and influencing local and systemic microenvironments^[4,5]^. Increasing evidence highlights their roles in both normal hematopoiesis and hematological malignancies^[6]^. Recent studies implicate EVs as critical contributors to MPN pathophysiology. EVs derived from malignant hematopoietic cells, including megakaryocytes, are found at elevated levels in MPN patient plasma and display altered cargo profiles compared to healthy controls^[7]^. These EVs promote a proinflammatory and prothrombotic microenvironment by delivering inflammatory cytokines, coagulation factors, and signaling molecules that activate endothelial cells, immune cells, and bone marrow stromal components, thereby fostering a niche supportive of clonal expansion and disease maintenance^[8]^. EVs derived from MPN cells have been shown to be enriched in proteins involved in oncogenic signaling pathways such as Janus kinase-Signal transducer and activator of transcription (JAK-STAT), phosphoinositide 3 kinase protein kinase B (PI3K-AKT), and nuclear factor kappa light chain enhancer of activated B cells (NF-κB), which promote proliferation, survival, and resistance to apoptosis in recipient cells. In addition, these EVs carry procoagulant factors such as tissue factor and phosphatidylserine, contributing to the heightened thrombotic risk in MPN patients^[7]^. However, the role of megakaryocyte-derived EVs in MPNs remains poorly understood. Characterizing their cargo may provide important insights into disease pathogenesis and holds promise for the identification of novel biomarkers for early MPN diagnosis.

The MEG-01 cell line, derived from a patient with megakaryoblast chronic myelogenous leukemia, exhibits megakaryocytic characteristics and platelet-forming capacity, making it a widely accepted *in vitro* model to study megakaryocyte biology and related hematopoietic processes^[9]^. Importantly, MEG-01 cells recapitulate key molecular features relevant to MPN pathology, including EV secretion with cargo reflective of megakaryocytic lineage and disease-associated signaling pathways^[10]^. In this study, we isolate high purity EV subtypes from MEG-01 cell culture medium using density gradient centrifugation and apply advanced mass spectrometry-based proteomics to comprehensively characterize their protein cargo. The objective of this study is to elucidate the protein cargos carried by the MEG-01 derived EV subtypes, thereby identifying novel biomarkers for MPN diagnosis and EV associated proteins contribute to MPN pathogenesis.

## METHODS

### Cell culture

MEG-01 cells, a human megakaryoblastic leukemia cell line, (ATCC® CRL-2021^TM^, RRID: CVCL_1405) were obtained from the American Type Culture Collection (ATCC, Manassas, VA, USA). Cells were cultured in RPMI 1640 medium supplemented with 2 mM L-glutamine (Gibco, Thermo Fisher Scientific, 25030081) supplemented with 10% (v/v) heat-inactivated fetal bovine serum (FBS; Gibco, A5256801) and 1% (v/v) penicillin-streptomycin (Gibco, 15140122). Cells were maintained at 37 °C in a humidified incubator with 5% CO_2_. Viability and cell count were monitored regularly using trypan blue exclusion with a hemocytometer. For EV collection, cells were cultured in serum free medium. Cells were incubated in serum free medium for 48 h prior to EV isolation. Cells were routinely tested and confirmed to be free of mycoplasma contamination using universal mycoplasma detection kit (ATCC, 30-1012K). Cell identity was authenticated by the ATCC through short tandem repeat (STR) profiling. All experiments were performed using cells with passage numbers below 30.

### EV isolation

EVs were isolated from 500 mL MEG-01 cell culture supernatant using a combination of differential ultracentrifugation and Nycodenz density gradient centrifugation as previously reported^[11]^. Conditioned medium was first subjected to sequential centrifugation at 4 °C to remove cells and debris: 300 × *g* for 10 min and 3,000 × *g* for 10 min. Crude large EVs (lEVs) were pelleted by centrifugation at 10,000 × *g* for 45 min. From the collected supernatant, crude small EVs (sEVs) were pelleted by ultracentrifugation at 100,000 × *g* for 2 h at 4 °C using an SW32Ti rotor (Beckman Coulter). The pellets were resuspended in phosphate buffered saline (PBS) and frozen at -80 °C until use, or 60% Nycodenz (Progen, 18003) for further purification using density gradient centrifugation. The EV pellets resuspended in 60% Nycodenz were added at the bottom of the gradient and layered with continuous Nycodenz gradient. The gradient was centrifuged at 39,000 rpm for 16 h at 4 °C using an SW41Ti rotor (Beckman Coulter). Twelve 1 mL fractions were collected from the top, and their densities were determined by refractometry. The refractive index of each collected gradient fraction was measured using a refractometer, and fraction density was calculated by converting refractive index values to density according to the manufacturer’s specifications for Nycodenz. Fractions with the presence of canonical EV markers were pooled, diluted in PBS (Gibco, 10010049), and subjected to a final ultracentrifugation at 100,000 × *g* for 2 h at 4 °C. The final EV pellets were resuspended in PBS and stored at -80 °C for downstream analyses.

### Western blotting

MEG-01 derived EVs were lysed in 1 × radioimmunoprecipitation assay buffer (RIPA) buffer (Cell Signaling, 9806) supplemented with 1 mM phenylmethylsulfonyl fluoride (PMSF), and protein concentration was determined using the micro-bicinchoninic acid (BCA) assay (Thermo Fisher Scientific, 23235). Equal amounts of EV protein or equal volume of fractions were mixed with Laemmli sample buffer. Proteins were separated on 10% sodium dodecyl sulfate-polyacrylamide gel electrophoresis (SDS-PAGE) gels and transferred to polyvinylidene difluoride (PVDF) membranes with 0.2 µm pore size (Thermo Scientific, 88520) using wet transfer at 110 V for 90 min at 4 °C. Membranes were blocked in 1% (w/v) fish skin gelatin in PBST (PBS + 0.1% Tween-20) for 30 min at room temperature, incubated overnight at 4 °C with primary antibodies, including mouse anti human CD41 (Thermo Fisher, BSM-33112M, clone 9B10, 1:1,000), mouse anti human CD63 (Thermo Fisher, 10628D, clone Ts63, 1:1,000), rabbit anti human CD81 (Cell signaling technology, 56039T, clone D3N2D, 1:500), mouse anti human HSP70 (Thermo Fisher, MA3-007, clone 5A5, 1:500), rabbit anti human HRS (Hepatocyte growth factor-regulated tyrosine kinase substrate; cell signaling technology, 15087T, clone D7T5N, 1:1,000), rabbit anti human Alix (Apoptosis-linked gene 2-interacting protein X; cell signaling technology, 92880T, clone E6P9B, 1:1,000) and mouse anti human Annexin A1 (Thermo Fisher, 66344-1-IG, clone 1E1B7, 1:500), diluted in blocking buffer, washed 3 times with PBST, and then incubated with goat anti rabbit (Invitrogen, 31460, 1:10,000) or goat anti mouse (Invitrogen, 31340, 1:10,000) HRP-conjugated secondary antibodies for 1 h at room temperature. Following three times wash in PBST, proteins were detected by enhanced chemiluminescence (Thermo Fisher, 34094) using a ChemiDoc imaging system (Bio-Rad).

### Transmission electron microscopy (TEM)

EVs were adsorbed onto Formvar/carbon-coated 200-mesh copper grids (Electron Microscopy Sciences, FCF200-Cu-50) by putting 20 µL of the sample on the grid surface for 10 min at room temperature. Excess liquid was carefully wicked off using filter paper. Grids were then fixed with 4% PFA in PBS (Thermo Fisher, J19943.K2) for 5 min to enhance structural preservation. Following fixation, grids were washed three times with deionized water to remove residual buffer salts. Negative staining was performed using 2% (w/v) aqueous uranyl acetate (Electron Microscopy Sciences, NC1085517) for 3 min. Stained grids were gently blotted to remove excess stain and allowed to air-dry completely at room temperature. Samples were imaged using a transmission electron microscope (T12) operated at an accelerating voltage of 120 kV.

### Nanoparticle tracking analysis (NTA)

Isolated EVs were diluted in 0.1 µm filtered PBS to obtain a particle concentration within the optimal range for analysis (1 × 10^8^ to 1 × 10^9^ particles/mL). Measurements were carried out using a NanoSight NS300 (Nanosight Technology, Malvern, UK). Instrument settings such as camera level, detection threshold, and blur settings were kept constant across all measurements. For each sample, three 30-s videos were recorded at room temperature, capturing particles moving under Brownian motion. Videos were analyzed using the manufacturer’s software (NTA version 3.1), and results were averaged across replicates. Data are reported as mean particle size ± standard deviation (SD) and particle concentration (particles/mL).

### sEV pull down

Protein A magnetic beads were coated with tetraspanin antibodies according to manufacturer’s protocol. Briefly, Dynabeads Protein A (Thermo Fisher, 10001D) were washed three times in PBS containing 0.1% bovine serum albumin (BSA) (Sigma-Aldrich, 05470-5G) to minimize nonspecific binding. Beads were incubated with primary antibody (mouse anti human CD9, clone HI9a, 312102, Biolegend; mouse anti human CD63, clone H5C6, 556019, Becton, Dickinson and Company (BD) Pharmingen; mouse anti human CD81, clone B11, sc-166029, Santa Cruz Biotechnology) at an indicated concentration of antibody per 50 µL beads with gentle rotation at room temperature. After 2 h incubation, unbound antibody was removed by using magnetic (Thermofisher, 12321D). The crude sEVs were added into the antibody coated beads and incubated with indicated period at 4 °C or room temperature. After incubation, the unbound material was removed using magnetic and collected for later using as flow through (FT). The unbound material was further removed by washing the beads 3 times with PBS. The pull-down (PD) material bound to beads was subsequently processed either for antibody labeling for flow cytometry or for western blot analysis using sample buffer.

### Cell flow cytometry

MEG-01 cells were collected and washed twice with PBS supplemented with 1% BSA and 0.02% NaN_3_ (Thermo Scientific, 190380050). Cell surface antigen staining was performed by incubating cells with fluorochrome-conjugated monoclonal antibodies, including CD9 (clone HI9a, 1:50, 312105, Biolegend). CD63 (clone H5C6, 1:50, 353007, Biolegend), CD81 (clone 5A6, 1:50, 349507, Biolegend), CD41/61 (clone PAC-1, 1:50, NBP2-62201, Novus Biologicals), CD40 (clone 5C3, 1:50, 334309, Biolegend), CD41 (clone HIP8, 1:50, 303709, Biolegend), CD42b (clone HIP1, 1:50, 303912, Biolegend), CD62p (clone AK4, 1:50, 304910, Biolegend), for 30 min on ice in the dark. Unstained and isotype-matched controls were included to enable proper gating and assessment of background fluorescence. After staining, cells were washed two times with cell staining buffer, resuspended in cell staining buffer and analyzed on the LSR Forteassa (BD) cytometer.

### EV MACSPlex

Surface molecule profiling of the isolated EVs was performed using the MACSPlex Exosome Kit (Miltenyi Biotec, 130-108-813) according to the manufacturer’s protocol. In brief, EV samples were prepared in PBS and adjusted to a protein concentration of 10 µg total EV protein per assay. EVs were incubated with a mixture of antibody coated capture beads. Bead-only controls (no EVs) were included to assess background signal and processed in parallel. After 16 h incubation at room temperature, beads were washed and subsequently stained with a fluorochrome-conjugated detection antibody cocktail targeting the tetraspanins CD9, CD63, and CD81. Samples were washed and analyzed by flow cytometry using LSR Fortessa (BD).

### Liquid chromatography tandem mass spectrometry analyses and data processing

Twenty micrograms of each sample were run on a NuPAGE 10% Bis-Tris gel (Invitrogen, NP0301BOX) for a short distance, the entire stained gel regions were excised and digested with trypsin. Liquid chromatography tandem mass spectrometry (LC-MS/MS) analysis was performed using a Q Exactive Plus mass spectrometer (ThermoFisher Scientific) coupled with a Vanquish Neo UHPLC system (ThermoFisher Scientific). Tryptic peptides were injected onto an Acclaim PepMapTM 100 trap column (75 μm i.d. × 2 cm packed with 3 μm C18 resin; ThermoFisher Scientific) and separated by reversed phase HPLC on a BEH C18 nanocapillary analytical column (75 μm i.d. × 25 cm, 1.7 μm particle size; Waters) using a 4-h gradient formed by solvent A (0.1% formic acid in water) and solvent B (0.1% formic acid in acetonitrile). Eluted peptides were analyzed by the mass spectrometer set to repetitively scan m/z from 400 to 1,800 in positive ion mode. The full MS scan was collected at 70,000 resolution followed by data-dependent MS/MS scans at 17,500 resolution on the 20 most abundant ions exceeding a minimum threshold of 10,000. Peptide match was set as preferred, exclude isotopes option and charge-state screening were enabled to reject unassigned, singly and > 6 charged ions.

Peptide sequences were identified using MaxQuant 1.6.3.3^[12]^. MS/MS spectra were searched against a UniProt human protein database (7/21/2022) and a common contaminants database using full tryptic specificity with up to two missed cleavages, static carbamidomethylation of Cys, and variable Met oxidation, protein N-terminal acetylation and Asn deamidation. “Match between runs” feature was used to help transfer identifications across experiments to minimize missing values. Consensus identification lists were generated with false discovery rates set at 1% for protein and peptide identifications. Statistical analyses were performed on protein label free quantification (LFQ) Intensity values using LFQ-Analyst^[13]^. Missing values were imputed with a minimum value, and p-values were adjusted to account for multiple testing using Benjamini-Hochberg false discovery rate (FDR) correction.

### Statistics

All statistical analyses were performed using GraphPad Prism (v8.0.2). Data are presented as mean ± SD unless otherwise stated. Statistical significance was determined using an unpaired two-tailed Student’s *t*-test for comparisons between two groups or one-way analysis of variance (ANOVA) followed by Tukey’s multiple comparisons test for comparisons among multiple groups. ns, not significant; ^*^P < 0.05; ^**^P < 0.01; ^***^P < 0.001. The proteomic data, including Gene Ontology (GO) annotation and Kyoto Encyclopedia of Genes and Genomes (KEGG) pathway enrichment analyses, was analyzed using Funrich (3.1.3). Flow cytometry data were analyzed using FlowJo (v10.10.1).

## RESULTS

### MEG-01 cells display platelet-associated markers and variable tetraspanin expression

MEG-01 cells cultured in full media exhibited robust proliferation, with cell density significantly increasing by day 2 compared to day 0, whereas cells maintained in serum-free media showed no appreciable growth over the same period, indicating that serum supplementation is essential for cell expansion [Figure 1A and B]. Despite the difference in proliferation, Annexin V/PI staining revealed that cell viability remained high under both culture conditions, with comparable proportions of live cells and no significant increase in apoptotic populations between full and serum-free media, suggesting that serum deprivation primarily affected proliferation rather than survival [Figure 1C and D]. To provide context for the subsequent characterization of MEG-01-derived EVs, we next examined the expression of canonical EV-associated tetraspanins and platelet-associated surface markers on parental MEG-01 cells. CD42b and CD62P were included because they are commonly reported markers of platelet-derived EVs and their expression on parental cells may influence their detection on the corresponding EV populations^[14,15]^. Flow cytometry analysis showed that MEG-01 cells expressed the classical tetraspanins CD63 and CD81, as well as the integrin complex CD41/CD61, whereas CD9, CD42b, and CD62P were not detected [Figure 1E]. Together, these findings demonstrate that while serum supplementation promotes cell proliferation, serum-free culture does not compromise cell viability and permits the recovery of EVs for marker profiling. Moreover, the use of serum-free conditions during EV collection eliminates contamination from serum-derived EVs and associated proteins, thereby facilitating the isolation of highly purified MEG-01-derived EVs for downstream characterization and proteomic analyses.

**Figure 1:**
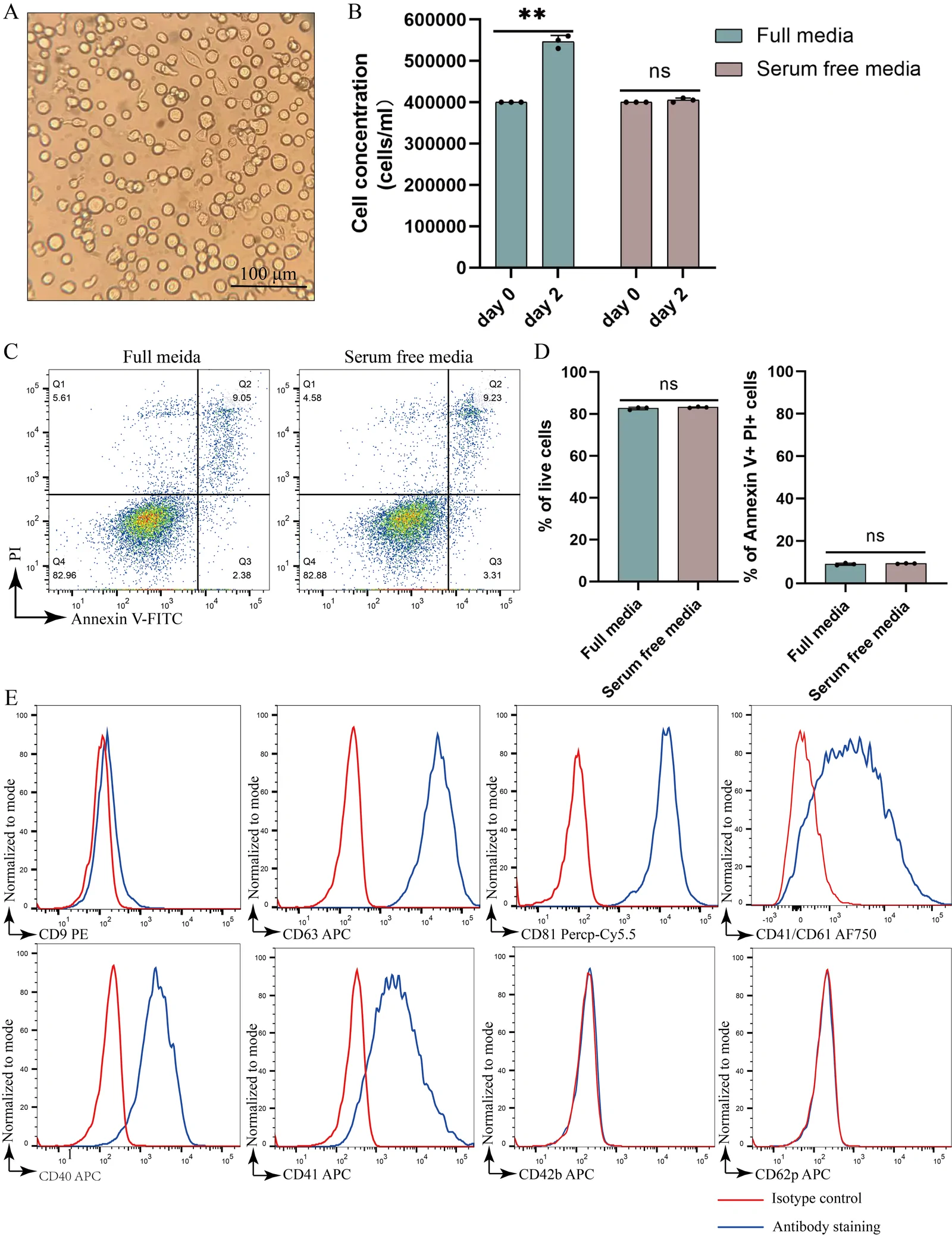
MEG-01 cells lack uniform expression of canonical EV tetraspanins, while maintaining platelet specific markers. (A) Representative bright-field microscopy image of MEG-01 cells culturing in serum-free media. Scale bar 100 μm; (B) Quantification of cell concentration at day 0 and day 2 for cells cultured in either full media (blue) or serum-free media (pink). Statistical significance was determined using an paired two-tailed Student’s *t*-test; *P* < 0.05. A significant increase in cell concentration was observed in full media, but not in serum-free media; (C) Flow cytometry dot plots showing Annexin V-FITC and PI staining of cells cultured for 2 days in full media or serum-free media; (D) Quantification of viable cells (Annexin V^-^/PI^-^; left) and apoptotic/necrotic cells (Annexin V^+^/PI^+^; right) from panel C, showing no significant difference between conditions; (E) Flow cytometry histograms showing the expression of surface markers CD9, CD63, CD81, CD41/CD61, CD40, CD41, CD42b, and CD62p on cells cultured in serum-free media. Blue histograms represent antibody staining and red histograms represent isotype controls. Representative bright-field images (A), flow cytometry plots (C), and flow cytometry histograms (E) from one of three independent biological experiments with similar results are shown. Quantitative data in (B and D) are presented as mean ± SD from three independent biological experiments. Statistical significance was determined using an unpaired two-tailed Student’s *t*-test. ns, not significant; ^***^P < 0.001. APC: Allophycocyanin; EV: extracellular vesicle; FITC: fluorescein isothiocyanate; PI: propidium iodide; PerCP-Cy5.5: peridinin-chlorophyll-protein complex-cyanine 5.5; SD: standard deviation; AF750: Alexa Fluor 750.

### MEG-01 cell derived EV isolation and characterization

EVs were isolated from MEG-01 cell culture supernatant through differential centrifugation as shown in Figure 2A, yielding a 10,000 × *g* fraction enriched in lEVs and a 100,000 × *g* fraction enriched in sEVs. Protein quantification showed that the lEV fraction contained approximately 1,100 µg/mL protein, significantly higher than the ~500 µg/mL obtained from the sEV fraction [Figure 2B]. SDS-PAGE and immunoblotting confirmed the enrichment of established EV markers, including the tetraspanins CD41, CD63, and CD81, as well as the chaperone HSP70 and membrane-associated Annexin A1 [Figure 2C]. NTA revealed distinct size distributions: lEVs exhibited a broader size profile with peaks around 300-500 nm, whereas sEVs were predominantly 100-150 nm in diameter, consistent with small vesicle populations. The mean particle size of sEVs (~150 nm) was significantly smaller than that of lEVs [Figure 2D]. TEM further confirmed the vesicular morphology of both fractions, with lEVs appearing as larger cup-shaped structures and sEVs as smaller, uniform, round vesicles [Figure 2E]. Flow cytometry analysis demonstrated successful detection of vesicles within the expected beads gate, with high proportions positive for the canonical EV markers CD63 and CD81 [Figure 2F]. Importantly, isotype control antibodies yielded no detectable signal above background, confirming that the observed fluorescence originated from specific antibody EV interactions rather than nonspecific binding. Surface protein profiling revealed differential marker enrichment across the vesicle populations: CD41, CD61, and CD81 showed the highest median APC fluorescence intensities, followed by CD9 and CD63, whereas other platelet-related proteins such as CD41b, CD42a, and CD62P were detected at lower but were present at substantially lower levels than the highly abundant markers [Figure 2G]. Analysis of the lEV surface molecules using the same approach shows that lEVs have similar surface molecule expression [Supplementary Figure 1]. Collectively, these results indicate the efficient isolation of both lEVs and sEVs from MEG-01 cells and demonstrate their distinct size distributions, conserved vesicular morphology, and expression of characteristic EV-associated proteins.

**Figure 2:**
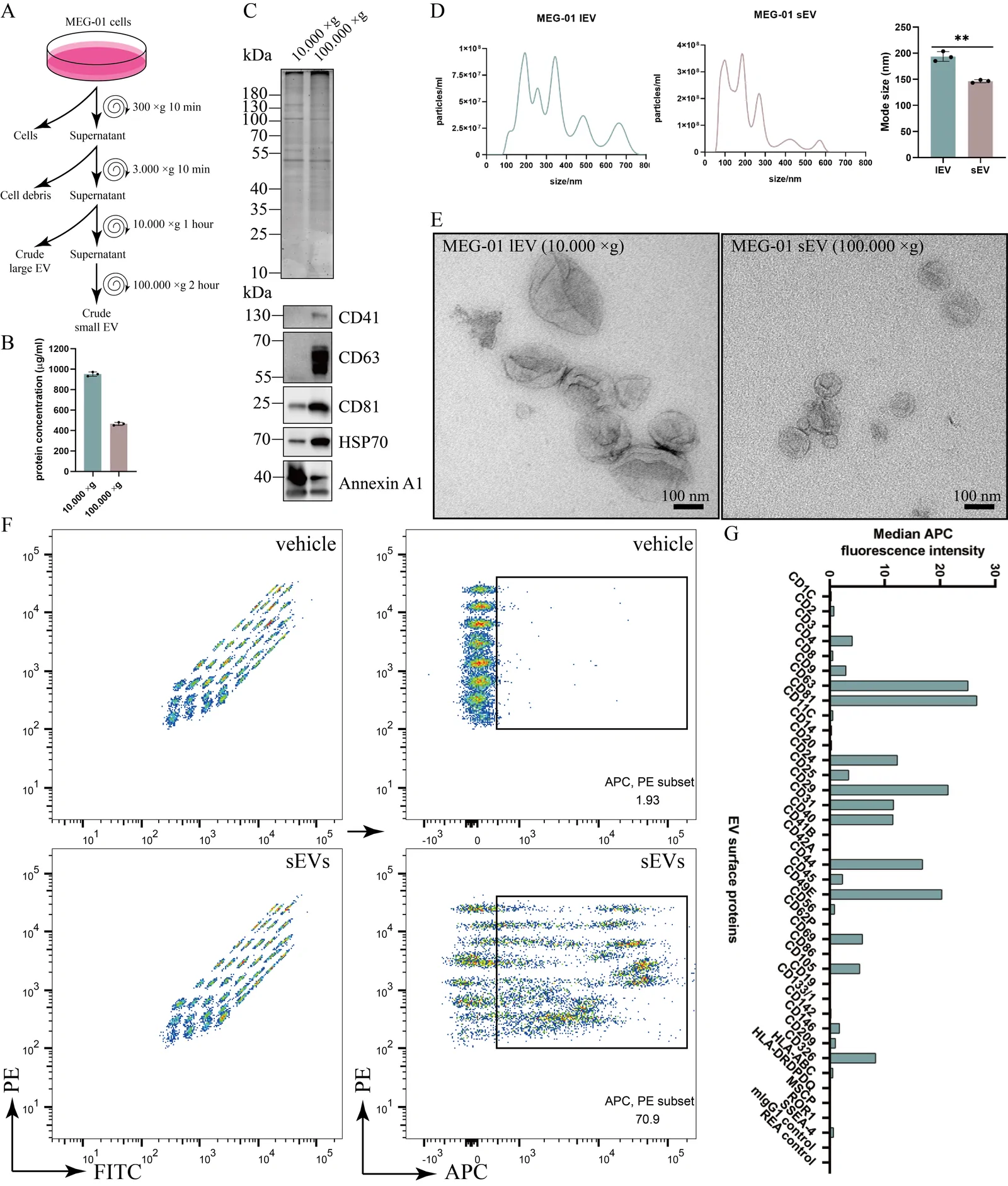
Isolation and characterization of lEV and sEV from MEG-01 cells. (A) Schematic of the differential centrifugation protocol used to isolate lEVs and sEVs from MEG-01 cell conditioned medium; (B) lEV and sEV were isolated from MEG-01 cell culture medium using differential centrifugation, and resuspended in the same volume of PBS. Quantification of total protein concentration in lEV and sEV preparations, as measured by BCA assay; (C) Same amount of protein as measured by BCA was loaded on the gel, and analyzed using SDS-PAGE and western blotting. SDS-PAGE gel (top) showing protein composition of EV fractions and western blot analysis (bottom) confirming the presence of canonical EV markers (CD41, CD63, CD81, HSP70, and Annexin A1) in MEG-01 EVs; (D) NTA profiles showing particle size distribution of large (left) and small (middle) EVs. Same settings were used when the particle size was measured using NTA. Bar graph (right) quantifies the modal size of lEV and sEV, revealing a significantly smaller size for sEVs; (E) TEM images of negatively stained lEVs (left) and sEVs (right). Scale bars = 100 nm; (F and G) MACSPlex analysis of surface molecules on the isolated MEG-01 sEVs. PBS was used as a vehicle control to assess non-specific binding of the detection antibodies. (F) Representative flow cytometry plots show clear staining for sEV surface molecules compared to vehicle control. The left panel shows successful detection of 37 EV-related antibody-coated beads, and the right panel shows successful detection of surface molecules on the EVs captured by each antibody-coated bead; (G) Quantification of the surface molecules as shown in (F). Representative immunoblots (C), NTA profiles (D), TEM images (E), flow cytometry plots (F), and MACSPlex fluorescence profiles (G) from one of three independent biological experiments with similar results are shown. Quantitative data in (B and D) are presented as mean ± SD from three independent biological experiments. Statistical significance was determined using an unpaired two-tailed Student’s *t*-test; ^**^P < 0.01. APC: Allophycocyanin; BCA: bicinchoninic acid; EV: extracellular vesicle; HSP70: heat shock protein 70; lEV: large extracellular vesicle; MACSPlex: multiplex bead-based assay for extracellular vesicle surface protein analysis; NTA: nanoparticle tracking analysis; PBS: phosphate-buffered saline; PE: phycoerythrin; SDS-PAGE: sodium dodecyl sulfate-polyacrylamide gel electrophoresis; SD: standard deviation; sEV: small extracellular vesicle; TEM: transmission electron microscopy.

### MEG-01 sEV PD using tetraspanin antibody-coated protein A beads

To examine the surface marker composition and tetraspanin heterogeneity of MEG-01-derived sEVs, we established an on-beads flow cytometry workflow for sEV surface protein profiling. Protein A magnetic beads were coated with anti-CD9, anti-CD63, or anti-CD81 antibodies and incubated with the 100,000 × *g* sEV fraction [Figure 3A]. To validate capture efficiency, PD and FT fractions were analyzed by SDS-PAGE, where total protein staining showed strong recovery of vesicle-associated proteins in the CD63 and CD81 PD fraction compared with FT, and immunoblotting confirmed robust enrichment of CD81 together with the EV markers Alix and HRS (Hepatocyte growth factor-regulated tyrosine kinase substrate) [Figure 3B]. To further assess antibody-dependent capture, we tested anti-CD63 coated beads across different incubation times (2, 4, and 6 h), and found that CD81 and HRS were strongly enriched in the PD fractions at all time points with corresponding depletion in FT, indicating stable capture efficiency over time [Supplementary Figure 2]. We next examined whether antibody-coated beads input influenced recovery efficiency by titrating increasing amounts of sEVs (2.5, 5, 10, and 20 µg) with anti-CD63 beads. Total protein staining and immunoblotting revealed consistent recovery of vesicle proteins in the PD fractions with proportional depletion in the FT, and robust enrichment of CD81, HRS, and Alix at all input levels [Supplementary Figure 3]. Similar results were obtained when the antibody-coated bead volume was varied, with CD81 and HRS remaining efficiently enriched across titrations [Supplementary Figure 4]. Control experiments using isotype antibody-coated beads resulted in minimal nonspecific binding, whereas anti-CD63 beads yielded robust PD signals with corresponding FT depletion [Figure 3C and D]. Together, these data demonstrate that antibody-coated beads enable efficient, scalable, and specific capture of MEG-01-derived sEVs, with enrichment efficiency maintained across incubation times, bead volumes, and vesicle input amounts, providing a validated workflow for downstream on-beads flow cytometry profiling for EV surface markers.

**Figure 3:**
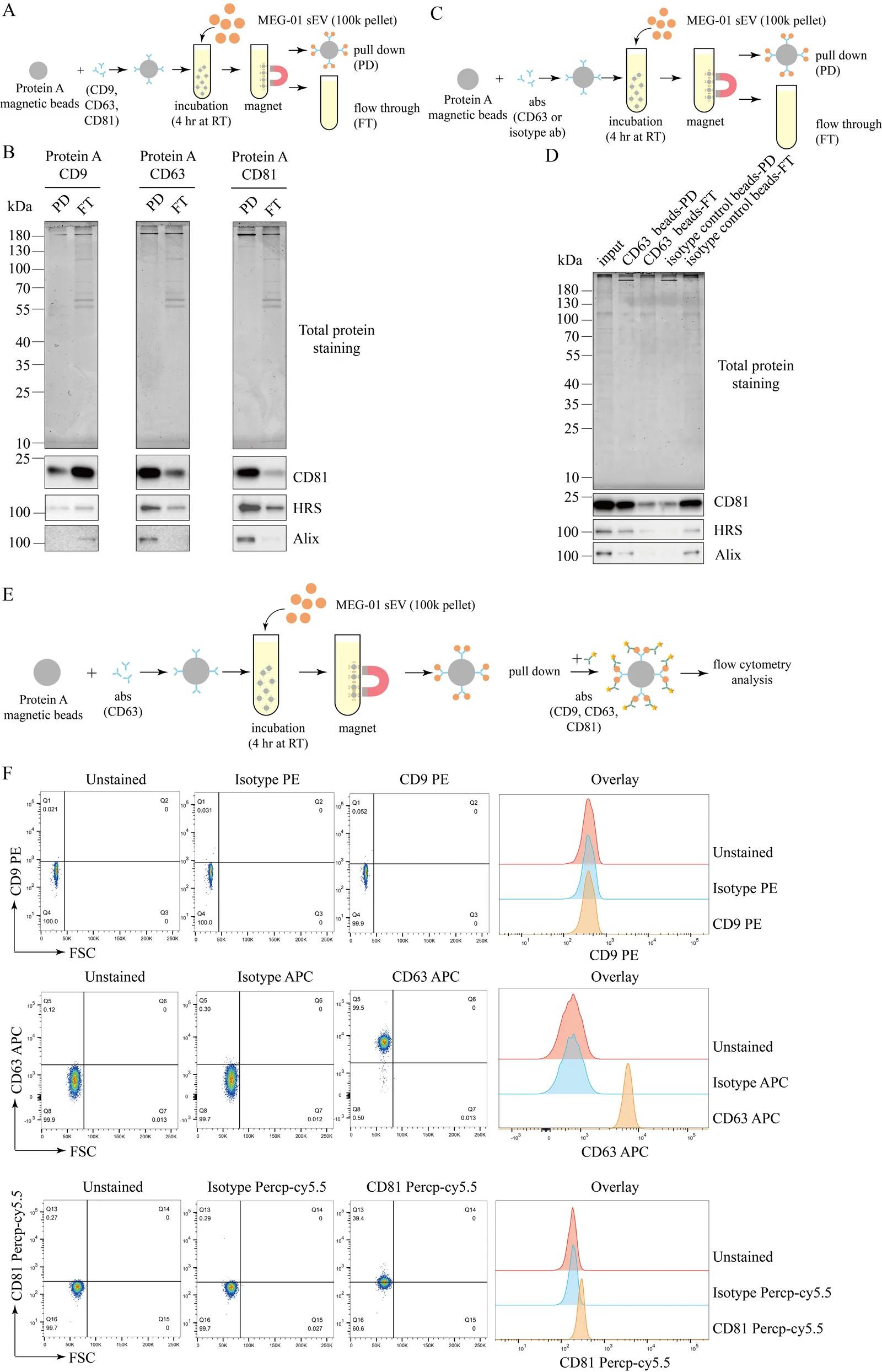
Detection of sEV surface molecules using on-beads flow cytometry. (A) Schematic workflow of immunoprecipitation of MEG-01 sEVs using Protein A magnetic beads conjugated to anti-CD9, CD63, or CD81 antibodies; (B) SDS-PAGE with total protein staining (top panels) and western blot analysis (bottom panels) of PD and FT fractions from immunoprecipitation using anti-CD9, CD63, or CD81. EV markers CD81, HRS, and Alix are shown; (C) Schematic of control immunoprecipitation using Protein A beads pre-bound with either anti-CD63 or isotype control antibody; (D) SDS-PAGE with total protein staining and western blot of PD and FT fractions from (C), confirming specific capture of CD63-positive sEVs; (E) Workflow of bead-based flow cytometry to analyze sEV surface markers (CD9, CD63, and CD81); (F) Representative flow cytometry plots of sEV PD using CD63-coated Protein A beads, labeled with antibodies against CD9 (PE), CD63 (APC), or CD81 (PerCP-Cy5.5), along with corresponding isotype and unstained controls, and detected using flow cytometry. Representative immunoblots (B and D) and flow cytometry plots (F) from one of three independent biological experiments with similar results are shown. APC: Allophycocyanin; EV: extracellular vesicle; FT: flow-through; HRS: hepatocyte growth factor-regulated tyrosine kinase substrate; PD: pull-down; PE: phycoerythrin; PerCP-Cy5.5: peridinin-chlorophyll-protein complex-cyanine 5.5; RT: room temperature; SDS-PAGE: sodium dodecyl sulfate-polyacrylamide gel electrophoresis; sEV: small extracellular vesicle.

In contrast to the efficient capture achieved using CD63-directed immunoisolation, CD9-mediated PD recovered only a limited subset of EVs. Most total protein content as well as the EV-associated proteins CD9, Alix, and HRS remained in the flow-through fraction [Supplementary Figure 5], indicating that a large proportion of MEG-01-derived sEVs are not readily captured through CD9 targeting alone. These findings highlight the heterogeneity of tetraspanin expression within the vesicle population and support the use of CD63 as the primary capture antibody for subsequent on-beads phenotypic analyses.

### On-beads flow cytometry to analyze MEG-01 sEV surface proteins

For multiparametric detection, sEVs were first captured with anti-CD63-coated beads and then stained on-bead with fluorophore-conjugated antibodies against CD9, CD63, and CD81 prior to flow cytometric acquisition [Figure 3E]. Gating on singlet bead events eliminated swarm artifacts and debris. Clear right-shifts in fluorescence histograms were observed for CD9-PE, CD63-APC, and CD81-PerCP-Cy5.5 relative to both unstained and isotype controls, yielding well-separated positive populations [Figure 3F]. Collectively, these data demonstrate that antibody-coated magnetic beads enable efficient, specific capture of MEG-01 sEVs and support sensitive, multiplexed flow cytometric detection of EV surface molecules, including canonical tetraspanins, directly on the bead-EV complexes.

### Purification of MEG-01 EVs using Nycodenz density gradient centrifugation

To achieve high-purity sEV isolation, pellets collected at 10,000 × *g* (lEVs) and 100,000 × *g* (sEVs) were further purified by Nycodenz density gradient ultracentrifugation^[11]^. Protein distribution across gradient fractions was first assessed by Sypro ruby total protein staining. Both lEV and sEV gradients showed protein bands spread over a wide range of fractions, indicating that multiple vesicular and non-vesicular proteins were present in the crude pellets prior to marker-based analysis [Figure 4A and B]. To distinguish fractions containing EVs, immunoblotting for specific EV markers was performed on the collected fractions. In the lEV preparation, Annexin A1, a marker associated with lEVs derived from plasma membrane budding, was most prominently detected in fractions 4-7 [Figure 4C], consistent with the expected density range (1.09-1.18 g/mL) of lEVs. In the sEV preparation, fractions collected from the sEV gradient were strongly enriched in the tetraspanins CD63 and CD81, which are widely recognized as canonical markers of endosome-derived sEVs, with the strongest signal observed in fractions 4-7 [Figure 4D]. These findings confirm that Nycodenz density gradient ultracentrifugation efficiently purifies lEVs and sEVs from crude preparations.

**Figure 4:**
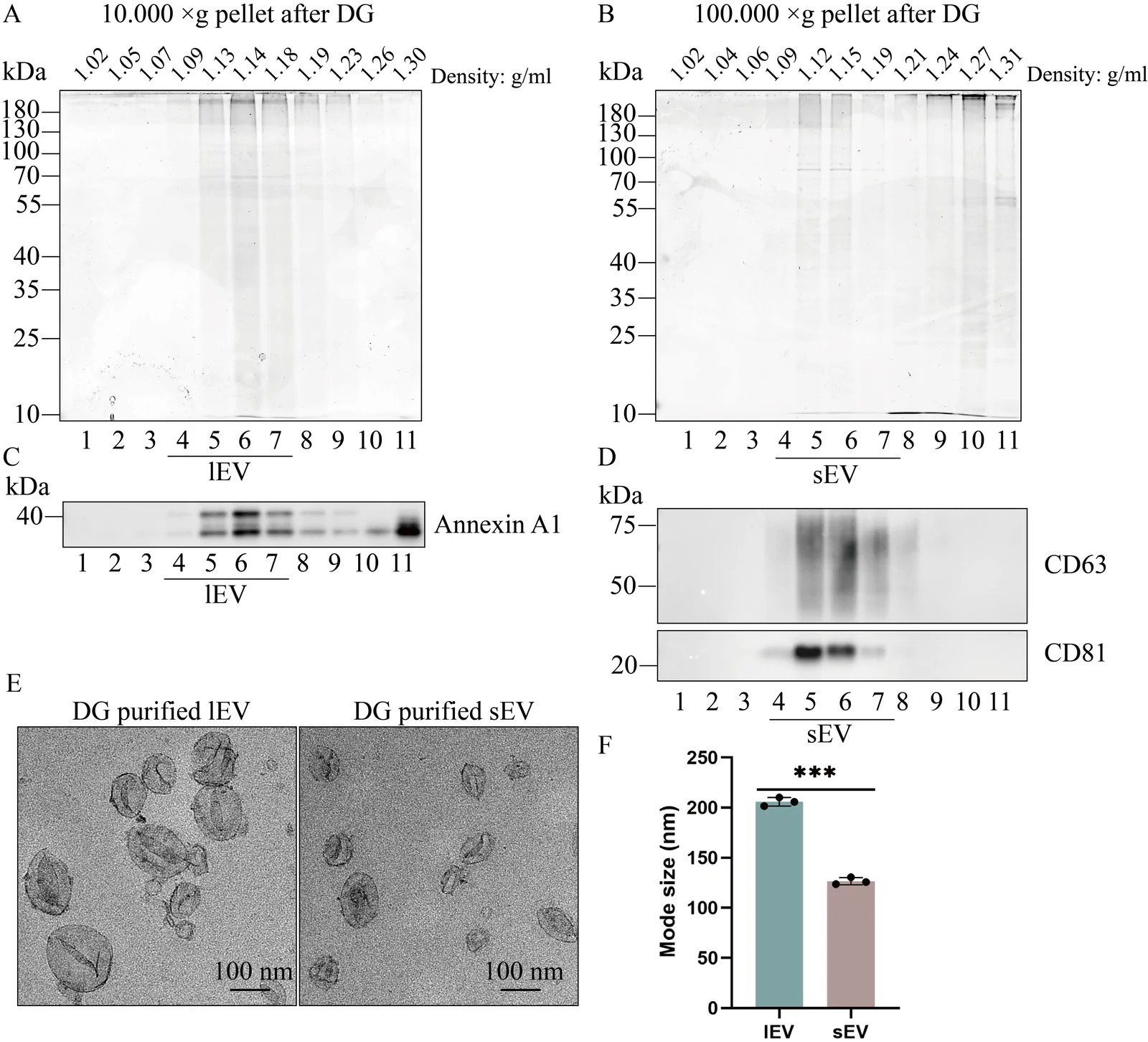
Characterization of Nycodenz DG-purified MEG-01 lEV and sEV. MEG-01-derived lEVs and sEVs were isolated from conditioned medium by differential ultracentrifugation and further purified by Nycodenz DG centrifugation to separate EVs from non-EV contaminants; (A) Total protein staining of density gradient fractions of 10,000 × *g* (lEV) pellet; (B) Total protein staining of density gradient fractions of 100,000 × *g* (sEV) pellet; (C) Western blot for Annexin A1, a lEV marker, shows enrichment of lEVs in the 4th-7th fractions; (D) Western blots for classical sEV markers CD63 and CD81 show enrichment in the 4th-7th fractions of the sEV gradient; (E) TEM images of DG-purified lEVs and sEVs reveal vesicular structures with EV-characteristic morphology. Scale bars: 100 nm; (F) Mode size distribution of purified lEVs and sEVs determined by NTA. Representative total protein staining (A and B), immunoblots (C and D), and TEM images (E) from one of three independent biological experiments with similar results are shown. Quantitative data in panel F are presented as mean ± SD from three independent biological experiments. Statistical significance was determined using an unpaired two-tailed Student’s *t*-test; ^***^P < 0.001. DG: Density gradient; EV: extracellular vesicle; lEV: large extracellular vesicle; NTA: nanoparticle tracking analysis; SD: standard deviation; sEV: small extracellular vesicle; TEM: transmission electron microscopy.

The morphology of density gradient-purified vesicles was subsequently examined by TEM. Representative images revealed intact, cup-shaped vesicles in both preparations, yet showed clear differences in size and morphology. LEVs appeared larger, more irregular in shape, and more heterogeneous in diameter, while sEVs displayed a smaller, more uniform morphology [Figure 4E]. Quantitative nanoparticle size distribution analysis corroborated these observations, demonstrating that sEVs exhibited a significantly smaller mode size compared with lEVs [Figure 4F]. Together, these results confirm the successful purification and characterization of lEVs and sEVs by density gradient ultracentrifugation, demonstrate their separation based on established protein markers, and highlight their distinct structural and size characteristics.

### MEG-01 lEVs and sEVs have a distinct proteome

To compare the protein composition of lEVs and sEVs, mass spectrometry-based proteomic analysis was performed [Supplementary Table 1]. Venn diagram analysis revealed a large overlap of identified proteins between lEVs and sEVs, with 4,664 proteins shared across both populations, while 103 and 275 proteins were uniquely detected in lEVs and sEVs, respectively [Figure 5A]. Importantly, 94 of the top 100 EV proteins listed in the ExoCarta database were detected in our dataset, confirming the high quality of EV preparations. Principal Component Analysis (PCA) demonstrated a clear separation between lEV and sEV proteomes along the first principal component, which accounted for 78.1% of the total variance, with replicates clustering tightly within each group [Figure 5B]. Unsupervised hierarchical clustering of significantly changing proteins further separated lEV and sEV samples into distinct clusters, indicating consistent differences in protein abundance patterns between the two EV subtypes [Figure 5C]. Differential expression analysis revealed a set of proteins significantly enriched in either lEVs or sEVs, as visualized in the volcano plot [Figure 5D]. Several cytoskeletal and membrane trafficking proteins were more abundant in lEVs, whereas proteins linked to RNA binding and vesicle-mediated transport were preferentially enriched in sEVs. Notably, multiple components of the RNA exosome complex, including EXOSC1, EXOSC2, EXOSC3, EXOSC4, EXOSC5, EXOSC6, EXOSC7, EXOSC8, and EXOSC9, were consistently and significantly enriched in sEVs compared with lEVs [Supplementary Figure 6]. All detected EXOSC subunits exhibited significantly higher abundance in sEVs, further supporting the enrichment of RNA-processing machinery within this EV subtype. Together, these findings demonstrate that while lEVs and sEVs share a large common proteome, they also display distinct protein signatures, underscoring their separation as functionally specialized EV subpopulations.

**Figure 5:**
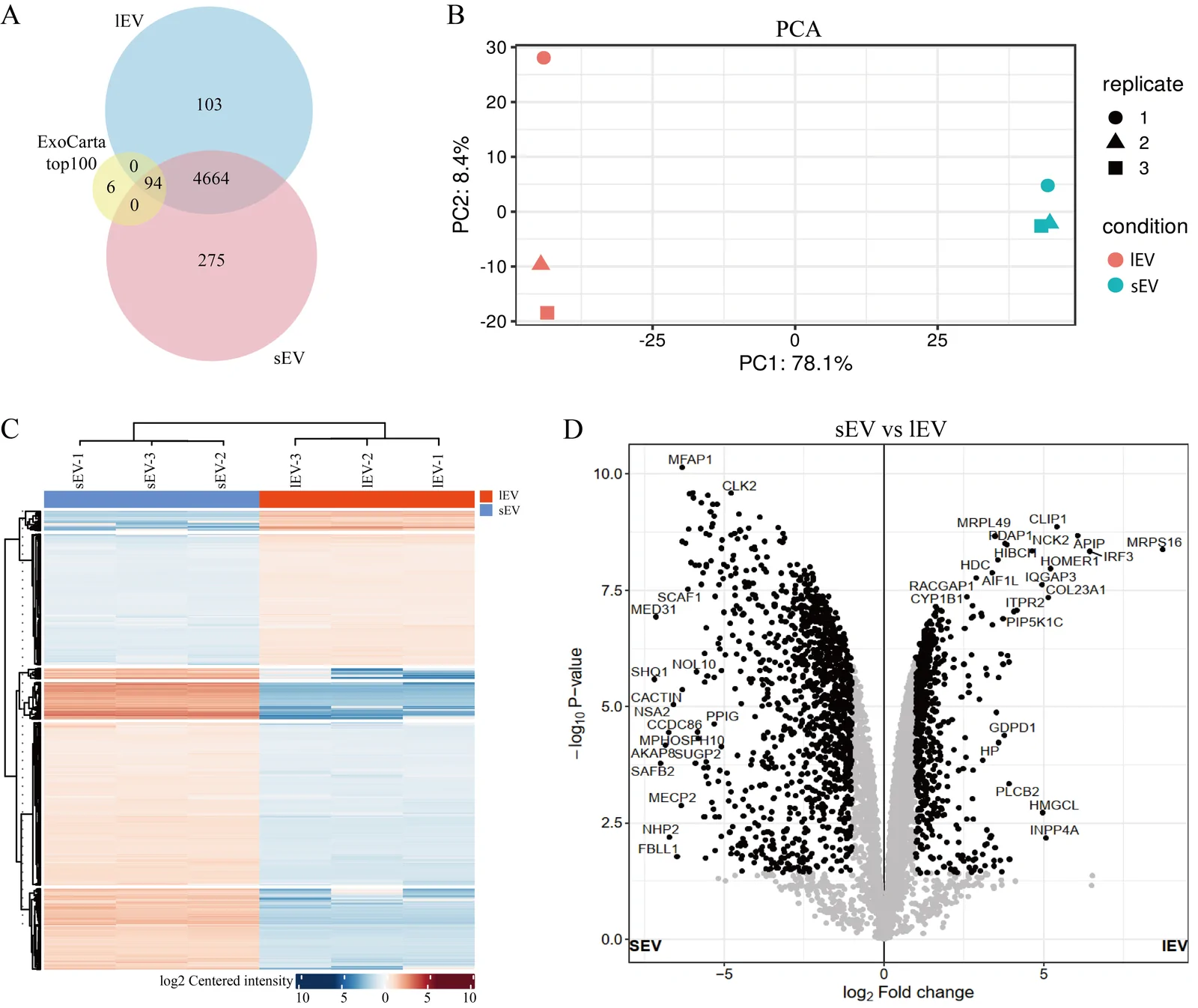
Proteomic comparison of DG-purified lEVs and sEVs from MEG-01 cells. (A) Venn diagram showing the overlap of proteins identified in lEVs, sEVs, and the top 100 proteins listed in ExoCarta; (B) PCA of proteomic profiles reveals clear separation of proteomes between lEVs and sEVs from MEG-01 cells. Each shape represents a biological replicate (*n* = 3); (C) Heatmap displaying the relative abundance of proteins detected in lEVs and sEVs; (D) Volcano plot comparing protein abundance between lEVs and sEVs. All proteomic analyses were performed using EVs isolated in three independent experiments for each group. PCA (B), heatmap (C), and volcano plot (D) were generated based on these biological replicates. DG: Density gradient; EV: extracellular vesicle; lEV: large extracellular vesicle; PCA: principal component analysis; sEV: small extracellular vesicle.

### GO and KEGG pathway enrichment of lEV and sEV associated proteins

To explore potential functional distinctions suggested by the proteomic profiles of MEG-01-derived lEVs and sEVs, we performed GO and KEGG pathway enrichment analyses on proteins identified in each EV subtype [Figure 6]. GO cellular component analysis revealed that proteins from both vesicle populations were predominantly associated with vesicle-related compartments, including exosomes, cytosol, plasma membrane, and organelle-associated structures. This indicates the high purity of the isolated lEVs and sEVs. Notably, sEVs contained a higher proportion of proteins annotated to the nucleolus compared with lEVs, indicating an enrichment of nuclear-associated components within sEV cargo [Figure 6A]. In Figure 6B, the majority of proteins in both lEVs and sEVs were annotated as “molecular function unknown.” Among proteins with defined molecular functions, the majority were associated with binding activities, including protein binding, RNA binding, and nucleic acid binding. Additional enriched functions included catalytic activity, representing enzymes involved in diverse metabolic and regulatory processes, as well as transporter and structural molecule activities. The distribution of proteins with defined molecular functions indicates that lEVs and sEVs have distinct molecular function profiles. In Figure 6C, proteins in both lEVs and sEVs were enriched in multiple biological processes, including signal transduction, cellular processes, metabolic pathways, and transport. A notable difference is that sEVs contained a higher proportion of proteins associated with RNA-related processes, whereas lEVs were relatively enriched in metabolic processes. Overall, these analyses indicate that lEVs and sEVs share many enriched biological processes but display distinct enrichment patterns across specific GO terms and KEGG pathways. KEGG pathway enrichment analysis further highlighted differences in pathway representation between vesicle subtypes [Figure 6D]. Proteins associated with lEVs were enriched in pathways related to proteasome function, metabolic processes, intracellular transport, and multiple infection- and stress-related pathways. sEV-associated proteins showed enrichment in pathways involved in DNA replication and repair, RNA processing and degradation, vesicle-mediated transport, and ribosome-related functions. Collectively, these analyses indicate that lEVs and sEVs derived from MEG-01 cells exhibit both shared and distinct protein cargo profiles, resulting in overlapping yet distinct GO and KEGG enrichment patterns that may reflect differences in their biogenesis and warrant further functional investigation.

**Figure 6:**
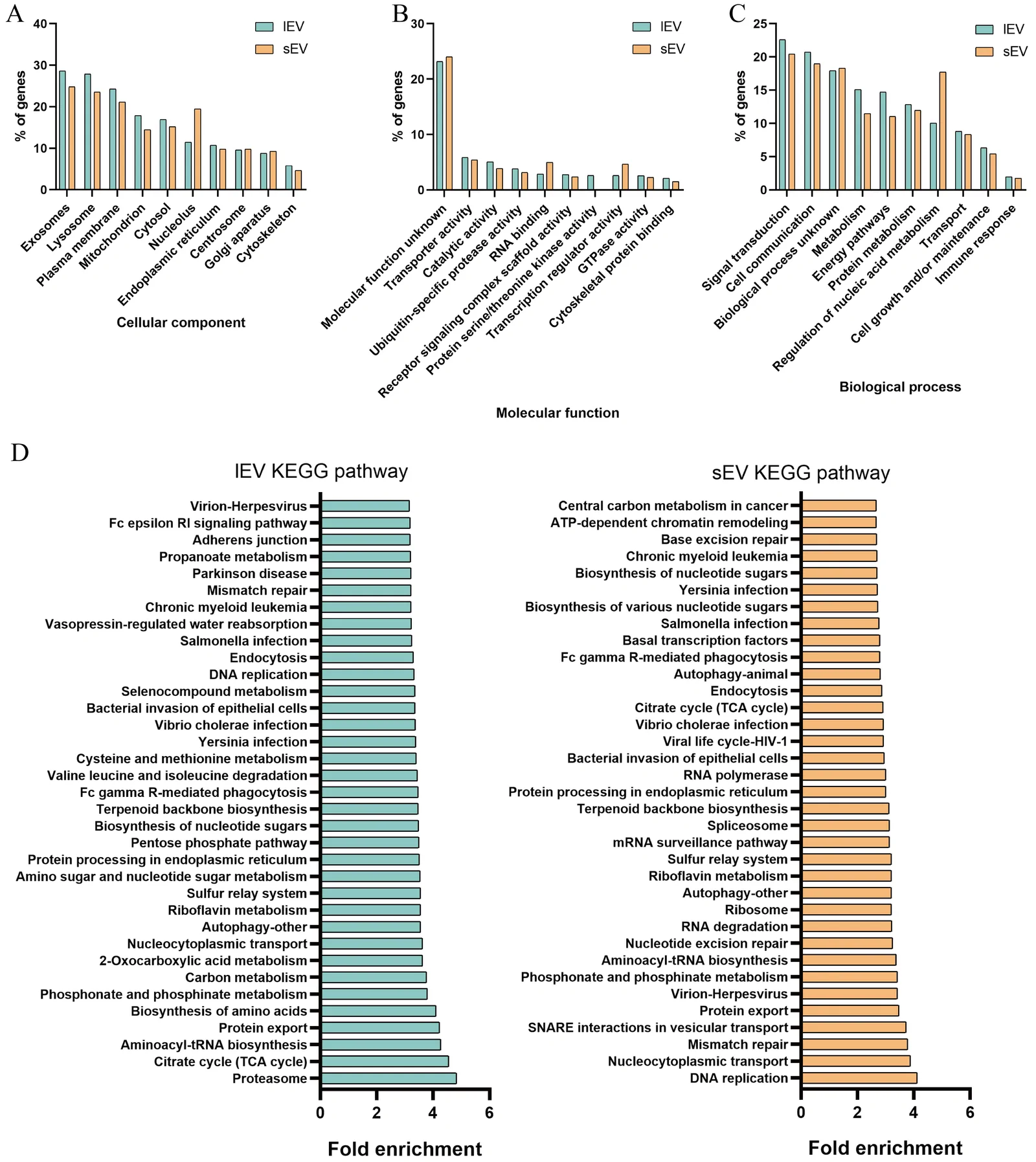
Comparative GO and KEGG pathway enrichment analysis of lEVs and sEVs derived from MEG-01 cells. (A-C) Comparative GO analysis of proteins identified in lEVs and sEVs isolated from MEG-01 cells. GO terms are grouped into the three main annotation categories: cellular component (A), molecular function (B), and biological process (C); (D) KEGG pathway enrichment analysis of proteins associated with lEVs (left) and sEVs (right) derived from MEG-01 cells. Enriched pathways are ranked according to fold enrichment, highlighting both shared and vesicle-type-specific pathways. For both GO and KEGG analyses, only proteins detected in all three biological replicates of each EV subtype were included. EV: Extracellular vesicle; GO: Gene Ontology; KEGG: Kyoto Encyclopedia of Genes and Genomes; lEV: large extracellular vesicle; sEV: small extracellular vesicle.

## DISCUSSION

EVs are emerging as key mediators in MPN, influencing inflammation, procoagulant activity, and the bone marrow microenvironment. EVs from megakaryocytes carry bioactive cargo that may contribute to disease progression and serve as potential biomarkers^[16]^. In this study, we delineate the heterogeneity of EV populations secreted by MEG-01 cells, identifying subtype-specific features in their size, structure, and proteome. The finding that MEG-01 cells maintain high viability under serum-free conditions demonstrates that these cells remain metabolically active during the EV collection period, supporting the use of serum-free culture for the isolation and characterization of MEG-01-derived EVs. This is consistent with the concept that EV release is a constitutive cellular process, not solely coupled to growth or proliferation^[17]^. Serum deprivation did not induce significant cell apoptosis, implying that the EVs recovered under these conditions are likely representative of physiologically relevant vesicles rather than products of stress-induced cell death. These data support serum-free culture of MEG-01 cells, which reduces contamination from bovine serum derived vesicles and allows the isolation of high purity EVs for downstream analyses^[18]^. Importantly, recent quantitative proteomic studies have shown that vesicles in 10k and 100k pellets from MEG-01 retain a conserved core of megakaryocyte- and platelet-associated proteins, while preserving features of an immature megakaryocytic state^[19]^. Together, these findings support the use of MEG-01 EVs as a model for EV biogenesis and cargo selection, while highlighting their differences from circulating platelet-derived EVs.

High purity EV isolation is essential in proteomic studies to ensure that identified proteins truly originate from vesicles rather than co-isolated contaminants. A previous study provides a comprehensive proteomic characterization of MEG-01 derived 10,000 × *g* and 100,000 × *g* pellets, including comparison with platelet- and plasma-derived EVs^[19]^. Consistent with increasing recognition in the EV field, differential centrifugation alone typically yields EV-enriched preparations that may still contain substantial amounts of non-vesicular material, including protein aggregates, lipoproteins, and other nanoparticles, which can influence downstream proteomic analyses^[11,20]^. Using differential centrifugation followed by density gradient ultracentrifugation, we achieved effective separation of lEVs and sEVs from co-pelleting non-vesicular material. High-density fractions contained substantial contaminating proteins, indicating that crude 10k and 100k pellets include significant non-EV components. These findings highlight the importance of density-based purification for accurate EV proteomic analysis. By combining density gradient purification with comprehensive characterization, we established a workflow for comparative analysis of high-purity lEV and sEV proteomes.

The use of antibody-coated magnetic beads for sEV capture revealed the robustness of Tetraspanin-based enrichment strategies. Interestingly, MEG-01-derived sEVs lacked detectable CD9, whereas CD63 and CD81 were highly abundant, consistent with the surface marker expression of parental MEG-01 cells.This observation aligns with previous reports showing that Tetraspanin expression on EVs can vary across different cell types^[21]^, reinforcing that canonical EV markers are not universally expressed. However, given the heterogeneous distribution of EV surface markers, antibody-based capture strategies inherently enrich only subpopulations defined by selected antigens and may therefore introduce selection bias. This marker dependent enrichment contrasts with density gradient based purification, which isolates vesicles according to their biophysical properties rather than selective surface recognition. To minimize subpopulation bias and enable unbiased proteomic profiling, we purified EVs by density gradient ultracentrifugation based on buoyant density rather than surface marker expression.

By analyzing the proteome of density gradient purified high purity EVs, we found that MEG-01 derived lEVs were enriched in cytoskeletal and membrane trafficking proteins, suggesting that these vesicles originate primarily from plasma membrane budding, consistent with classical biogenesis pathways^[22]^. In contrast, sEVs were enriched in RNA-binding proteins and components associated with vesicle-mediated transport, supporting an endosomal origin^[23]^. Together, these data indicate that subtype-specific functional architecture becomes evident only after high-purity isolation removes non-vesicular background signals. These results suggest that MEG-01 cells produce molecularly and functionally distinct EV subtypes, with lEVs potentially contributing to structural remodeling and intercellular adhesion, and sEVs mediating RNA- and protein-based intercellular signaling.

Proteomic analysis further emphasized functional specialization between MEG-01 derived lEVs and sEVs. While a large core proteome was shared, each vesicle type carried unique proteins enriched in biologically meaningful pathways. lEVs were enriched in proteins involved in cytoskeletal organization and membrane trafficking, reflecting their plasma membrane origin. In contrast, sEVs were enriched in RNA-binding proteins, components of the spliceosome, and ribosomal proteins, indicating a potential role in modulating RNA metabolism and protein synthesis in recipient cells. This suggests that sEVs may be specialized for intercellular communication, possibly transferring functional RNA and protein cargo to influence gene expression in target cells. Although further studies are required to define the RNA content of sEVs, these findings support a model in which EV subtype diversity enables megakaryocytes to engage in multifaceted signaling within their microenvironment.

Our proteomic analysis of MEG-01-derived EVs revealed a broad set of proteins with potential relevance to MPN pathogenesis. Cytoskeletal and adhesion proteins such as PLEC, FLNA, TLN1, ACTN1/4, MYH9/10, and SPTAN1 were enriched in EVs, consistent with their role in platelet and megakaryocyte biology^[24,25]^. The presence of these proteins suggests that EVs may facilitate abnormal cell-cell and cell-matrix interactions, promoting megakaryocyte expansion and contributing to bone marrow fibrosis, a defining feature of advanced MPN. Importantly, cytoskeletal proteins carried by EVs could also enhance platelet activation and adhesion to endothelium, thereby exacerbating the prothrombotic risk that characterizes MPN patients. In addition, EV cargo was enriched in proteins involved in RNA regulation, metabolism, and vesicle trafficking, including POLR2A/B, SF3B1, SRSFs, HK1/2, LDHA, FASN, IDH1/2, CLTC, RABs, and PDCD6IP. The presence of these proteins within EVs suggests a role in horizontal transfer of regulatory molecules to hematopoietic and stromal cells, thereby shaping the bone marrow microenvironment. For instance, EV-mediated delivery of RNA-binding proteins or metabolic enzymes could alter gene expression programs or metabolic states in recipient cells, fostering clonal dominance of malignant hematopoietic progenitors^[26]^. These pathways are consistent with the notion that EVs act as messengers of malignant signaling, extending the impact of megakaryocytes beyond their direct proliferation. Importantly, proteins linked to DNA repair, signaling, and proteostasis were detected in MEG-01 EVs. Their transfer could contribute to genomic instability, survival signaling, and stress adaptation in neighboring cells, potentially driving disease progression and leukemic transformation in MPNs^[27]^.

Beyond their pathogenic role, the consistent enrichment of canonical EV proteins alongside MPN-relevant functional cargo highlights the biomarker potential and disease-monitoring of MEG-01 EVs. Platelet- and megakaryocyte-associated surface proteins reflect the abnormal megakaryopoiesis central to MPN pathogenesis, and their presence in circulating EVs could serve as a surrogate measure of aberrant megakaryocyte activity and platelet overproduction, providing a minimally invasive readout of disease burden^[28]^. Such EV-based markers could complement current hematological parameters (e.g., platelet counts and bone marrow morphology) while offering higher specificity for disease-associated vesicle release. In this study, we developed an on-bead method to specifically and efficiently pull down EVs and detect EV surface molecules. Future validation in patient cohorts will be essential to determine the specificity and sensitivity of these candidate markers, but our findings strongly support the exploration of megakaryocyte EV proteins as biomarkers for MPN diagnosis and clinical monitoring.

While our findings provide new insights into the molecular composition and heterogeneity of MEG-01 derived EVs, several limitations should be acknowledged. Our analyses were performed using the MEG-01 cell line, which offers a useful model for investigating megakaryocyte derived EVs but may not fully capture the complexity of EV populations in MPN patients. In addition, although density gradient centrifugation enabled the enrichment of highly purified lEV and sEV, EV populations remain heterogeneous and cannot be completely separated into discrete subtypes. The biological implications of the distinct protein cargo identified in lEVs and sEVs were inferred primarily from proteomic profiling and pathway enrichment analyses. While these findings suggest potential differences between EV subtypes, further studies will be needed to determine how these vesicles influence recipient cells and contribute to disease associated processes. Future investigations using primary patient samples and functional validation will help to further define the biological and clinical significance of megakaryocyte derived EV heterogeneity in MPN.

In summary, we established a workflow for isolating highly pure MEG-01-derived EV subtypes and demonstrated their molecular heterogeneity and functional specialization. These findings provide insights into EV-mediated communication and protein trafficking in MPN, support the discovery of EV-based biomarkers for diagnosis, and offer a framework for investigating EV-mediated mechanisms in megakaryocyte-driven hematological disorders.

## DECLARATIONS

### Availability of data and materials

The raw data supporting the findings of this study are available within this Article and its Supplementary Materials. Further data are available from the corresponding authors upon request.

### AI and AI-assisted tools statement

During the preparation of this manuscript, ChatGPT (version 5.2, released 2026-04-09) was used solely to assist in generating the initial graphical abstract. The AI tool was used only for generating the initial graphical abstract and did not influence the study design, data collection, data analysis, interpretation of the results, or the scientific content of the work. All authors reviewed, edited, and approved the final graphical abstract and manuscript and take full responsibility for the accuracy, integrity, and final content of the work.

### Copyright

© The Author(s) 2026.
