# Supplementary material for: Proteomic profiling of high purity extracellular vesicle subtypes from megakaryoblastic leukemia cells provides insights into myeloproliferative neoplasm biology
Source: Extracell Vesicles Circ Nucl Acids. 2026 Sep 2;7(3):1369–87. doi: 10.20517/evcna.2026.38 (PMC13673345; doi:10.20517/evcna.2026.38)
Supplement: Supplementary file 1 [file evcna-7-3-1369-SupplementaryMaterials.zip › evcna7038-SupplementaryMaterials/evcna7038-SupplementaryMaterials.pdf]

Supplementary Materials

Proteomic profiling of high purity extracellular vesicle subtypes from megakaryoblastic leukemia cells provides insights into myeloproliferative neoplasm biology

Xiaogang Zhang[1], Sarita Sehra[2], Cynthia Timmers[2], Tony Chadderton[2], Matthew Stubbs[2], Xiaowei Xu[1]

[1]Department of Pathology and Laboratory Medicine, University of Pennsylvania, Philadelphia, PA 19104, USA.
[2]Incyte Research Institute, Wilmington, DE 19803, USA.

Correspondence to: Prof. Xiaowei Xu, Department of Pathology and Laboratory Medicine, University of Pennsylvania, Philadelphia, PA 19104, USA. E-mail: xug@pennmedicine.upenn.edu

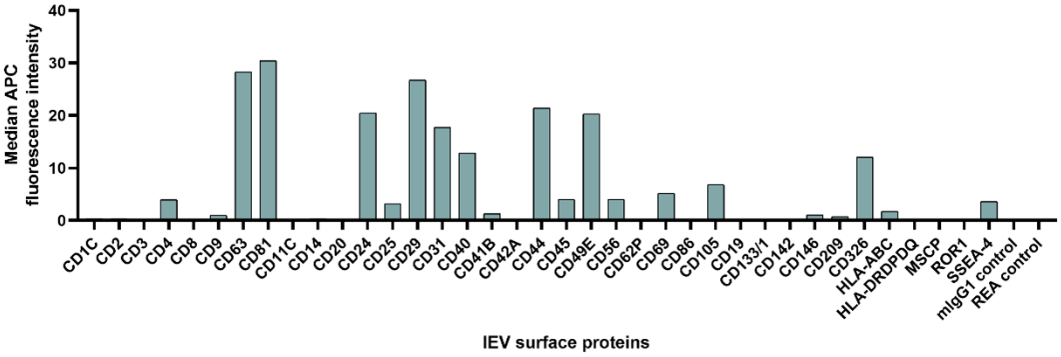

Supplementary Figure 1. MACSPlex analysis of lEV surface molecules. lEVs were isolated from MEG-01 cell culture media and their surface molecules were analyzed using MACSPlex. The profile shown is representative of one of three independent biological experiments with similar results.

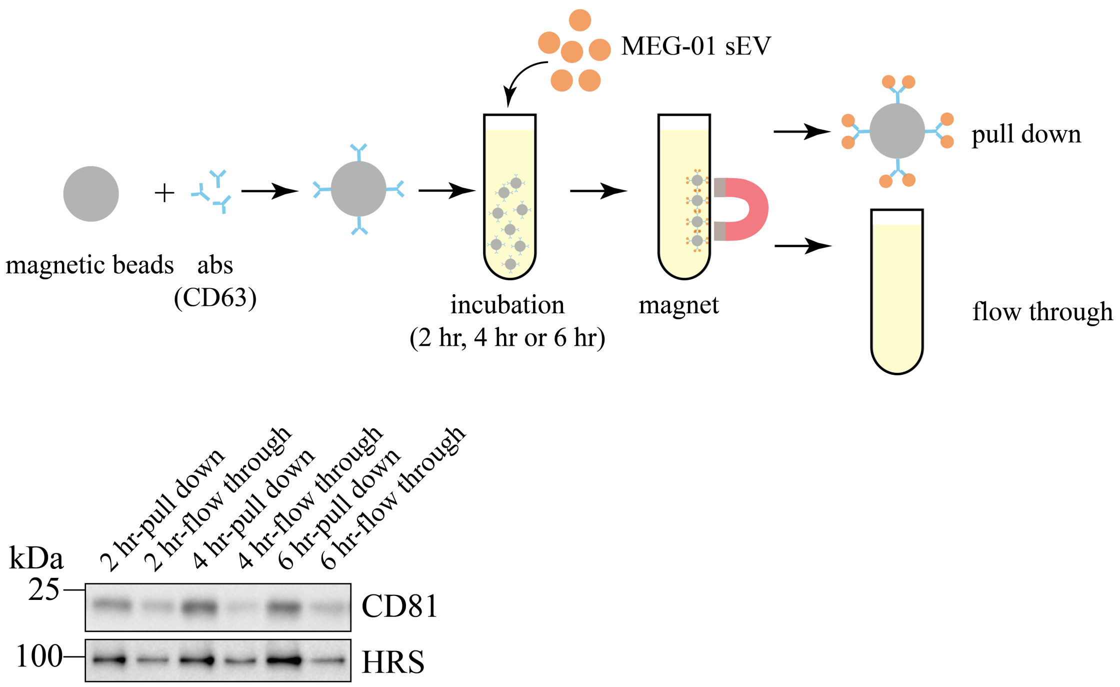

Supplementary Figure 2. Optimization of incubation time for efficient capture of MEG-01 sEVs using CD63-coated protein A beads. To assess the effect of incubation duration on sEV recovery, 10 µg of MEG-01-derived sEVs were incubated with 100 µL of CD63 antibody-coated protein A magnetic beads at room temperature for 2, 4, or 6 hours. Following incubation, bead-bound and flow-through fractions were analyzed by western blotting for the presence of sEV markers CD81 and HRS. Extended incubation times led to increased recovery of sEV markers in the bead-bound fraction, indicating improved capture efficiency. Representative immunoblots are shown from one of three independent biological experiments with similar results.

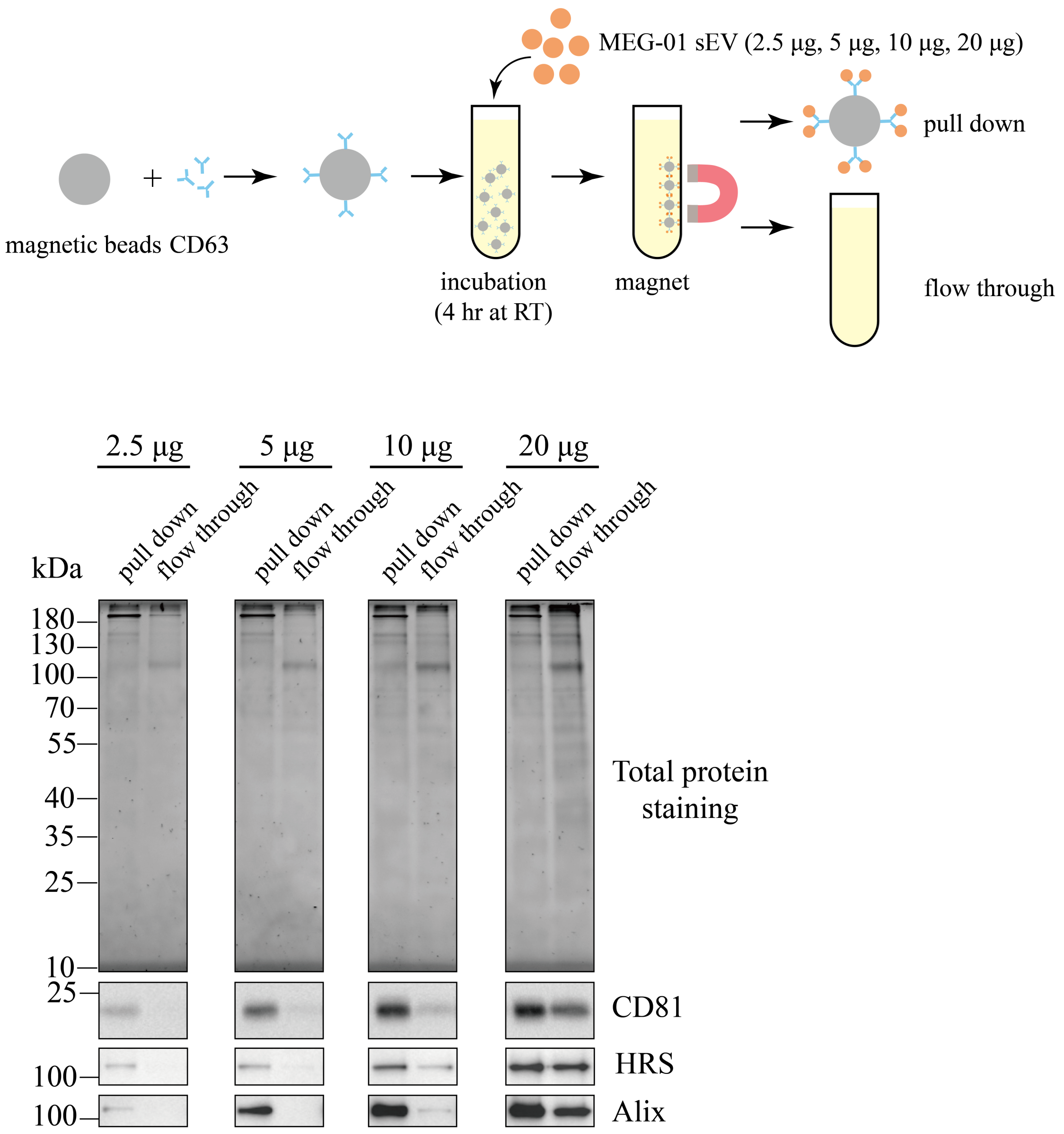

Supplementary Figure 3. Evaluation of the binding capacity of CD63-coated protein A beads for MEG-01 sEVs. To determine the maximum amount of sEVs that can be captured, increasing amounts of MEG-01-derived sEVs were incubated with 100 µL of CD63 antibody-coated protein A magnetic beads for 4 hours at room temperature. Bead-bound and flow-through fractions were analyzed by total protein staining using SYPRO Ruby staining and by western blotting for sEV markers CD81, HRS, and Alix. Saturation of bead binding capacity was indicated by decreased marker enrichment in the bead-bound fraction and increased signal in the flow-through at higher sEV input levels. Representative immunoblots are shown from one of three independent biological experiments with similar results.

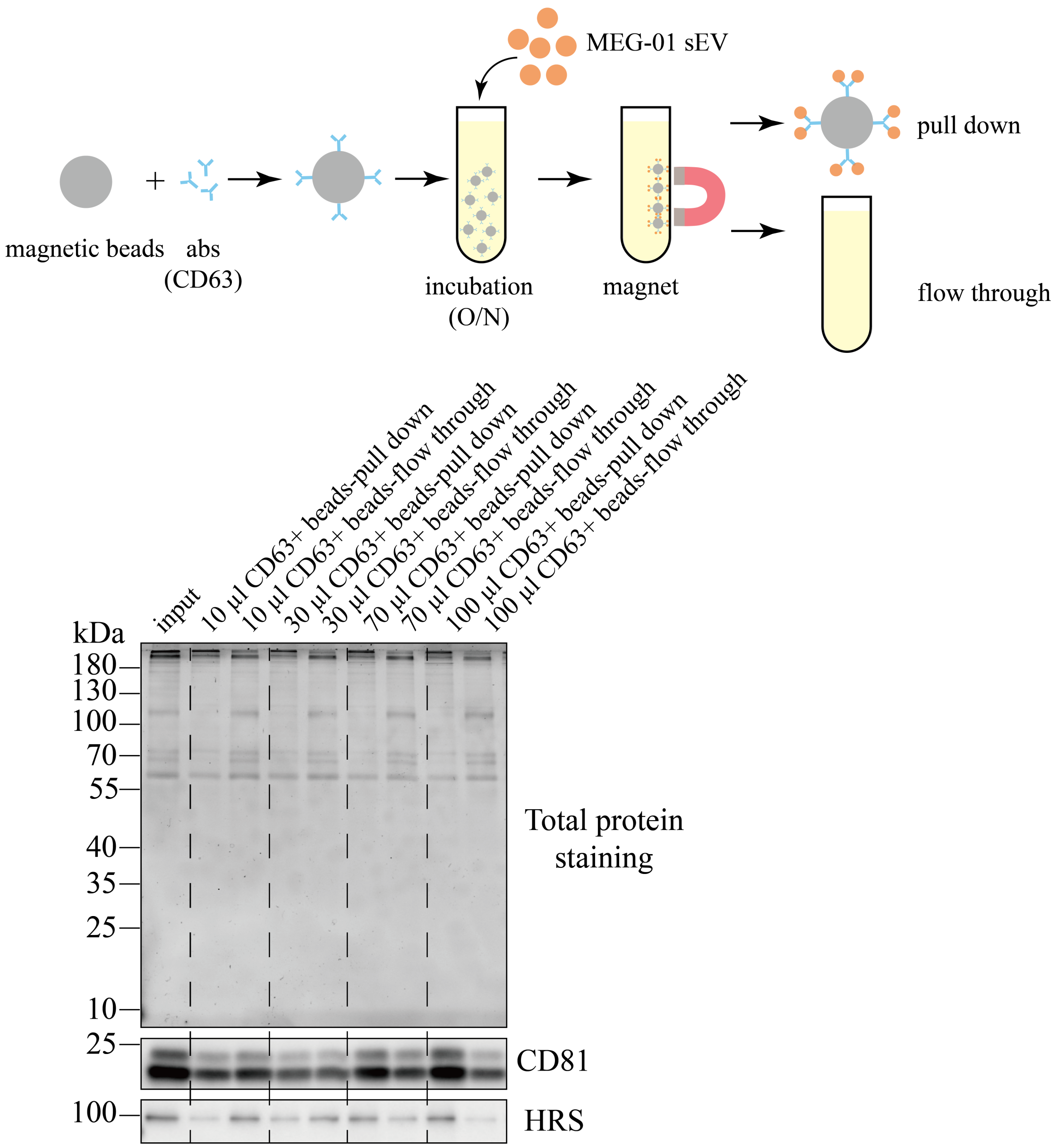

Supplementary Figure 4. Optimization of CD63 antibody coating for efficient immunoprecipitation of MEG-01 sEVs. Protein A magnetic beads were coated with increasing amounts of CD63 antibody to determine the optimal conditions for sEV capture. A fixed amount (10 µg) of MEG-01-derived sEVs was incubated with the antibody-coated beads overnight. The bead-bound and flow-through fractions were subsequently analyzed by SDS-PAGE with Sypro Ruby staining and western blotting for sEV markers CD81 and HRS. Efficient enrichment of sEVs was observed with increasing antibody amounts, as indicated by the enhanced recovery of protein and marker signals in the bead-bound fraction. Representative immunoblots are shown from one of three independent biological experiments with similar results.

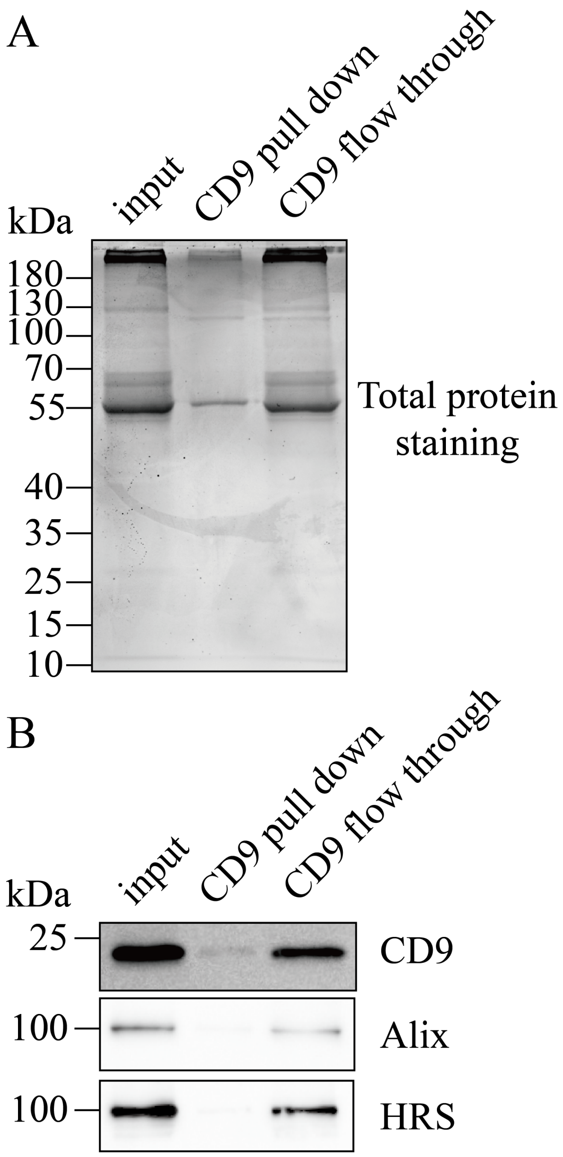

Supplementary Figure 5. CD9-coated protein A beads do not efficiently capture MEG-01-derived sEVs. sEVs isolated from the 100,000 × *g* pellet were subjected to immunoprecipitation using CD9-coated protein A magnetic beads. The pull-down and flow-through fractions were normalized to same volume, analyzed by SDS-PAGE followed by total protein staining with SYPRO Ruby (A), and by western blotting (B) to detect sEV markers CD9, Alix, and HRS (B). Minimal protein and marker detection in the pull-down fraction indicate that CD9-coated protein A beads fail to efficiently isolate MEG-01 sEVs under the tested conditions. Representative immunoblots are shown from one of three independent biological experiments with similar results.

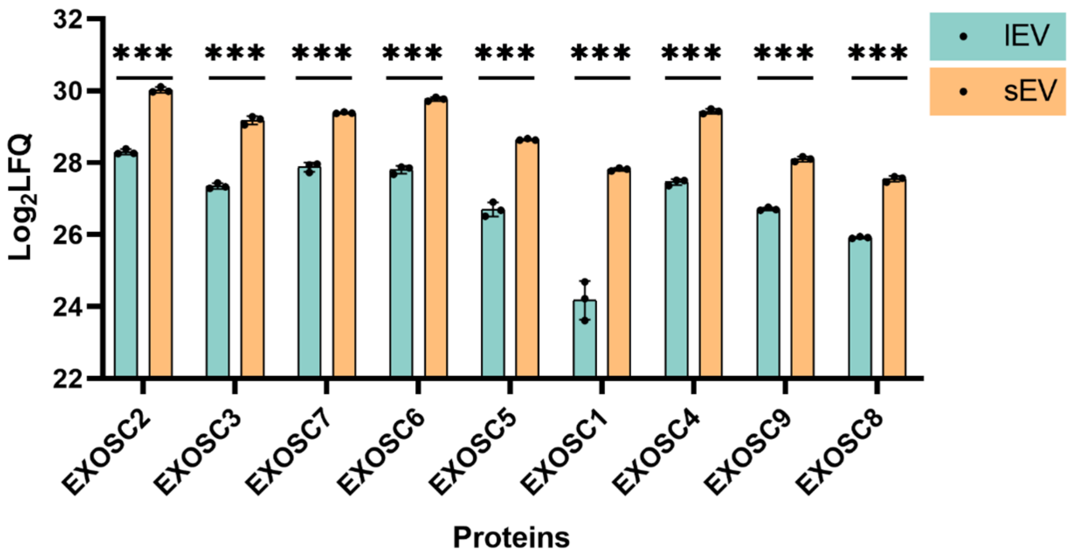

Supplementary Figure 6. Comparative analysis of exosome complex components in density gradient purified MEG-01 lEV and sEV by LC-MS. Equal amounts of total protein from density gradient purified lEVs and sEVs derived from MEG-01 cells were analyzed by LC-MS. The relative abundance of detectable exosome complex components was compared between the two EV subtypes. Differences in the representation of these components reflect potential variation in EV biogenesis and cargo sorting mechanisms.
